# *From Someone Who May Cause Trouble to Someone You Can Play With*: Stakeholders' Perspectives on Preschool Program Quality for Autistic Children

**DOI:** 10.1007/s10803-021-05268-2

**Published:** 2021-09-09

**Authors:** Hampus Bejnö, Sven Bölte, Nina Linder, Ulrika Långh, Samuel L. Odom, Lise Roll-Pettersson

**Affiliations:** 1 Department of Special Education, Stockholm University, 106 91 Stockholm, Sweden; 2 Division of Neuropsychiatry, Department of Women’s and Children’s Health, Center of Neurodevelopmental Disorders (KIND), Karolinska Institutet, Stockholm, Sweden; 3 Center for Psychiatry Research, Stockholm County Council, Stockholm, Sweden; 4 Autism Center for Young Children, Habilitation & Health, Stockholm County Council, 104 62 Stockholm, Sweden; 5 Frank Porter Graham Child Development Institute, University of North Carolina at Chapel Hill, CB 8180, Chapel Hill, NC 27599-8180 USA; 6 Child and Adolescent Psychiatry, Stockholm County Council, Stockholm, Sweden; 7 Curtin Autism Research Group, Curtin School of Allied Health, Curtin University, Perth, WA Australia; 8 KIND, CAP Research Center, Gävlegatan 22 B, 11330 Stockholm, Sweden

**Keywords:** Autism, Learning environment, Preschool, Program quality

## Abstract

In Sweden, young autistic children typically attend community-based preschool programs, which may not be adapted to their needs. In the current study, stakeholders to autistic children receiving Early Intensive Behavioral Intervention were interviewed following a quasi-randomized study (#NCT03634761) aimed at improving the preschool program quality using the Swedish version of the Autism Program Environment Rating Scale (APERS). Stakeholders provided their perceptions and experiences concerning key factors for high quality preschool programs as well as well as their experiences of the abovementioned APERS study. Applying thematic analysis, stakeholder groups differed in what they emphasized, but all highlighted staff’s competence, children’s inclusion and participation, collaboration, and the learning environment as key program areas that had been positively influenced by the APERS-based intervention.

Autism is a neurodevelopmental condition characterized by altered social interaction and communication, as well as repetitive, stereotypical behaviors and restricted interests [American Psychiatric Association (APA), 2013]. According to figures from Maenner et al. (2020) about 1 out of 54 children in the USA are diagnosed with autism by 8 years of age, although other estimates indicate a prevalence of about 1% in high-income countries, with estimates of between 1 and 2% in the Stockholm region of Sweden (Fombonne et al., 2021; Idring et al., 2015; Lord et al., 2020). These factors in combination with the fact that almost all children in Sweden attend preschool [National Center for Education Statistics (NCES), 2017; Sveriges Kommuner och Landsting (SKL), 2018] including autistic children, are placing high demands on the Swedish support system to provide early intervention of high quality (Magnusson et al., 2014). In Sweden, following a clinical diagnosis, early interventions for young autistic children may be provided as either focused interventions, or comprehensive intervention programs [i.e., Early Intensive Behavioral Intervention (EIBI)].

Focused interventions target single, individualized goals over a limited amount of time (i.e., until the goal is achieved) (Steinbrenner et al., 2020). In contrast, comprehensive programs encompass a variety or series of focused interventions targeting a broad range of skills over several domains such as communication, language, daily living skills, and peer-interaction. As of date EIBI is typically regarded as the comprehensive program with most research support (Eldevik et al., 2009; Reichow et al., 2018).

EIBI is a systematic and structured approach for teaching preschool children socially significant behaviors (Klintwall & Eikeseth, 2014), and typically targets skill domains that may be challenging for the autistic child (e.g., language, imitation, communication). A range of evidence-based practices are used, both in preschool settings, and provided by children’s parents at home (Eldevik et al., 2019). Positive reinforcement is used to promote new skills, interventions are individualized and supervised by competent and trained professionals, data is continuously collected to inform goal settings, choosing of teaching procedures, and to evaluate progress (Eldevik et al., 2019). There is research indicating that EIBI can impact developmental trajectories of autistic children; leading to significant improvement in adaptive behaviors, IQ-scores, communication, social, cognitive skills, as well as reduction of the severity of specific autistic traits (Eldevik et al., 2009, 2012; Flanagan et al., 2012; Matson & Konst, 2014; National Autism Center, 2015; Perry et al., 2013; Warren et al., 2011; Wong et al., 2015). However, in order for this to occur in mainstream preschool settings, EIBI of high quality needs to be implemented with fidelity (Långh et al., 2020). In such programs, trained staff support the autistic child over the course of the day and provide opportunities to interact with typically developed peers who may also serve as role models (Strain & Bovey, 2011). Additional features of high quality learning environments also include the competence and credentials of supervisors’ and preschool teachers, low teacher–child ratio, and minimal staff turnover (Eldevik et al., 2019; Scheuermann et al., 2003). Other factors likely to affect the quality of learning environments are staff buy-in, and support from leadership (Odom et al., 2019).

In regard to EIBI in Sweden, preschool principals usually employ paraprofessionals to work specifically with the autistic child in the preschool (part of education system). If parents choose and in collaboration with the preschool program, regionally funded habilitation centers (part of health care system) provide EIBI workshops and weekly or bi-weekly supervision to preschool staff (i.e., mainly paraprofessional) and parents. These services are usually provided at the habilitation center (Roll-Pettersson et al., 2016). This form of service involves groups of stakeholders, including preschool principals, preschool paraprofessionals and teachers, as well as parents and habilitation supervisors. In sum, implementation of EIBI is based on a dualistic systemic collaboration, i.e., administered by habilitation and implemented in preschool and home settings. Consequently, the EIBI system in Sweden somewhat differs from EIBI systems in the U.S., where state health departments are responsible for early intervention for children below 3 years of age, while older children (i.e., 3–21) are administered through school districts [Centers for Disease Control and Prevention (CDC), 2021]. According to Swedish habilitation recommendations (Föreningen Sveriges Habiliteringschefer, 2012) EIBI programs should start as early as possible, with a duration of 2 years, a minimum of 25 h of weekly instruction in both preschool and home settings, at least 5 days a week, with the majority of instruction provided in the preschool and supervision at a habilitation center. This service format is similar to parallel forms of service provided to young children with autism in other well-resourced countries (e.g., U.S., U.K.; CDC, 2021).

However, obstacles hampering the quality of implementation of evidence-based intervention in Sweden have been noted. For example, Långh et al. (2017) found a low level of acceptance among preschool staff for implementing evidence-based practices based on the principles of applied behavior analysis (ABA), as well as limited knowledge about evidence-based practices for autistic children. In addition, a study by Zakirova-Engstrand and Roll‐Pettersson (2014) indicated neutral to negative attitudes towards inclusion of autistic children among preschool staff. Furthermore, in an ethnographic case study by Roll-Pettersson et al. (2016), the authors identified three barriers to implementation of high quality EIBI. First, paraprofessionals receiving EIBI supervision at habilitation center tend to become isolated, with very limited involvement of other preschool staff in implementation. Second, interviewed stakeholders (i.e., parents, habilitation supervisors, and preschool staff) had major concerns about the lack of knowledge and skills concerning autism among preschool staff in general. Third, differing theoretical frameworks and guidelines between habilitation and preschool negatively affected implementation of early intervention. Also, in a report from the Swedish School Inspectorate (Skolinspektionen, 2017), only 30% of a random sample of 35 Swedish preschools were deemed as providing adequate support to children with special education needs, which is concerning given that the national Swedish curriculum (Skolverket, 2018) strongly emphasize the importance of support and participation for children in need of special support. Moreover, although there is a lack of sound data, the information at hand indicate that staff turnover is increasing in the Swedish preschool (Sveriges Radio, 2018). Furthermore, a recent report from the Swedish Ministry of Education and Research (Utbildningsdepartementet, 2020) describes a dire need for increased knowledge about neurodevelopmental conditions among educational staff, proposing the incorporation of modifications in Swedish higher education programs for preschool- and school teachers, as well as special educationalists. Taken together, these prevailing systemic shortcomings within the Swedish support system may negatively affect the quality of the learning environment, and thereby broadening the developmental gap between autistic children and typically developing peers.

In an effort to implement and evaluate a professional development model designed to improve overall preschool and school program quality for autistic children, the Autism Program Environment Rating Scale (APERS) was developed by the National Professional Development Center on Autism Spectrum Disorder in the US (NPDC; Odom et al., 2013) to assess the quality of educational programs for autistic children (Odom et al., 2018). The APERS for preschool and elementary school consists of 59 items, scored on a 5-point Likert-scale from 1 (poor quality) to 5 (high quality). Items are grouped into the 10 following domains: Learning Environments, Positive Learning Climate, Assessment and Individual Education Plan (IEP) Development, Curriculum and Instructions, Communication, Social Competence, Personal Independence, Functional Behavior, Family Involvement, and Teaming. It has demonstrated a high level of overall internal consistency (α = 0.94–0.96), with all domains loading to one factor, conceptually defined as program quality (Odom et al., 2018). This information can be used to develop an action plan for improving the program quality for autistic children.

The theory of change by the NPDC operates on the assumption that the quality of an educational program serves as a platform or foundation, on which evidence-based practices can be implemented (Odom et al., 2013). Following this assumption, the APERS can be used to assess and improve preschool- or school program quality for autistic children, providing the supportive context for implementing evidence-based practices with fidelity. These practices can be embedded in EIBI programs, which in combination may produce positive outcomes for autistic children (Odom et al., 2013). Applying such a model, with APERS assessments and feedback combined with in-service training and on-site coaching (Kucharczyk et al., 2012), produced significant improvements of preschool- and school program quality as well as autistic students’ goal attainment in 132 preschool- and school intervention programs for autistic children in 12 states in the USA (Odom et al., 2013). Furthermore, in an additional evaluation over 60 public elementary schools, including 486 autistic students, improvements were made in the learning environments for the inclusive educative programs, as well as for children’s goal attainment (Sam et al., 2020).

With a basis in previously described shortcomings within the Swedish system, and finding from studies conducted by Odom et al. (2013) and Sam et al. (2020), to complement EIBI with an environmentally focused intervention, Bejnö et al. (2021) conducted a preregistered (#NCT03634761) and nonequivalent pretest–posttest group study (Portney & Watkins, 2015). In the study, the translated, culturally adapted and validated Swedish version of the Autism Program Environment Rating Scale (APERS-P-SE; Bejnö et al., 2019) was used to enhance preschool program quality for autistic children. The study involved a total of 17 preschools that included one child with autism who received EIBI. All preschools were community-based (i.e., tax subsidized municipality or independent preschools) and represented a variety of socioeconomic conditions. Eight of the preschools were independent preschools, while the other nine preschools were municipal preschools. Nine preschools were assigned to the experimental group and eight preschools to the control group. Children in both groups received the EIBI program (i.e., with weekly- or bi-weekly EIBI supervision mainly provided at a habilitation center). In the experimental group, the APERS-P-SE was conducted, the preschool staff received the results and one and a half day of in-service training on autism and learning environment, the preschool staff and habilitation supervisors developed action plans for improving preschool learning environment for autistic children, supervisors from the habilitation center and preschool staff received a one and a half day of in-service training on the NPDC model and coaching, and habilitation supervisors provided monthly on-site coaching with the involvement of at least two preschool staff.

Project duration was approximately 8 months, and the results showed that the targeted preschools displayed significant improvements in total preschool program quality for autistic children compared to the control condition, specifically in the areas of learning environments, and personal independence and competence, and that participating children also displayed high levels of goal attainment. The preschool staff in the experimental group completed a social validity rating scale based on Wolf’s (1978) conceptualization of social validity at the end of the study, which indicated high ratings of goals, procedures and outcomes (Bejnö et al., 2021). However, to the authors best knowledge there are no previously published studies conducted within the Swedish educational context aimed at understanding stakeholders’ perception of high-quality preschool practice and EIBI for autistic children, and subsequently no reported qualitative outcomes on participants’ experiences of participating in research aimed at improving preschool program quality. Gathering qualitative information is important, as it may provide a deeper understanding of stakeholders’ experiences (Cleland, 2017) beyond quantitative outcomes of intervention effects. Understanding lived experiences can be valuable to understand EIBI intervention settings, to enrich the understanding of how the APERS-based-model was perceived, and provide directions for future systemic improvements.

The aims of the current study were to gain insight from stakeholders (i.e., parents to autistic children, preschool principals, preschool staff, and habilitation supervisors) about how they experienced the APERS-P-SE-based model as well as its importance for students and preschool staff.

This study addresses two research questions:

1. What do stakeholders perceive as key factors for providing a high-quality preschool program for autistic children who are receiving EIBI to promote optimal development?
2. How do stakeholders perceive that the APERS-P-SE-based intervention influenced the preschool program quality for the autistic child?

## Method

### APERS-P-SE-Based Model

Within the framework of the current study, the APERS-P-SE was applied as an addition to regular EIBI provision as follows. First, the scale was used to assess strengths and weaknesses of the quality of the learning environment for autistic children in all 17 preschools (see Bejnö et al., 2021). Following assessment, feedback was provided to two preschool staff in each preschool. Second, following group-assignment, preschool staff in the experimental group were coached to construct an action plan, based on APERS-P-SE information, focusing on improving the learning environment. Also, APERS-P-SE feedback was provided to habilitation supervisors in the experimental condition (APERS-P-SE preschools). Preschool staff who had previously received feedback (typically, the assigned paraprofessional and a preschool teacher) from each preschool participated in preparatory in-service training concerning inclusion, participation, naturalistic evidence-based practices, learning environment for autistic children, and goal scaling. Furthermore, habilitation supervisors (i.e., clinical psychologists, speech- and language pathologists, and special education teachers) who already supervised the children’s EIBI programs received equivalent preparatory in-service training. Subsequently, the preschool staff received monthly one-hour on-site coaching sessions from the habilitation supervisor responsible for the children’s EIBI program. Coaching focused on inclusive learning goals, implementing evidence-based practices to promote learning and inclusion, and to improve overall quality of the learning environment based on the initial APERS-P-SE ratings. Midway through the intervention, preschool staff participated in a follow up-seminar to discuss and reflect on their work in promoting the preschool program quality, as well as the children’s development. In addition, supervisors in the APERS-P-SE preschools group participated in two workshops; one at onset of study on coaching (see Kucharczyk et al., 2012), and midway through the project on inclusive evidence-based practices (e.g., incidental teaching and peer-mediation). Thus, to summarize, all participating preschools provided EIBI programs to the children who participated in the study. For the preschools in the experimental group, the prevailing child-focused EIBI program was complemented with the environment-focused APERS-P-SE-based model, with the primary aim of improving preschool learning environment quality for autistic children. Detailed descriptions of the formats, contents, and structure are provided in Table 1.

**Table 1:** APERS-P-SE-based model formats, structure and contents

Format	Description	Objectives	Duration
APERS-P-SE assessment	Ratings of overall preschool program quality, domains, subdomains and individual items by collecting information through observations, interviews and reviews of documentation, by the study’s first author	To identify key areas of strength and weaknesses in the preschool program for children with autism	6–7 h
APERS-P-SE-based feedback on learning environment	Written and oral feedback on areas of strength and weakness provided to preschool staff following APERS-P-SE assessment, by the study’s first author	To function as the base for professional development, and to guide systematic improvement of preschool program quality for children with autism through in-service training and on-site coaching	30–60 min
In-service training	A one-day workshop in the basic tenets of APERS-P-SE and features of high-quality preschool programs, selected evidence-based practices, goal scaling, and how to promote children’s inclusion and engagement	To prepare the preschool staff for formulating goals, receiving on-site coaching, promote their knowledge about autism and evidence-based practices, to improve important features of their preschool programs for children with autism	7 h
Initial coaching session(s)	Collaborative development of action plan between habilitation supervisor (i.e. “coach”) and preschool staff. This entailed identifying, formulating and scaling preschool goals and children’s goals based on initial APERS-P-SE feedback and to select relevant evidence-based practices to implement	To develop an action plan entailing goals on child and preschool level, to prepare for future coaching sessions in order to improve overall preschool program quality for children with autism	1–2 h
Following coaching sessions	Coaching sessions with habilitation supervisor (i.e. “coach”) and preschool staff including a pre-observational meeting, observations and post-observation meetings, and the use of implementation checklists	To provide support in implementing evidence-based practices to promote children’s goal attainment, and to improve other quality aspects of the preschool program such as the physical set-up of the environment	1 h per sessions
Midway-seminar	Midway-seminar for participating preschool staff. It included repetition on selected evidence-based practices, discussions about progress made, joint problem-solving and troubleshooting in collaboration with other preschool staff, and members of the research team, on how to make further improvements in preschool program quality for children with autism	To follow up participating preschool staff and their work in improving their preschool program quality for children with autism. To provide additional support for the preschool staff in their work, and to further establish a community of practice	4 h
Following coaching sessions	Coaching sessions with habilitation supervisor (i.e. “coach”) and preschool staff including a pre-observational meeting, observations and post-observation meetings, and the use of implementation checklists	To provide support in implementing evidence-based practices to promote children’s goal attainment, and to improve other quality aspects of the preschool program such as the physical set-up of the environment	1 h per session
Final coaching session	Habilitation supervisor (i.e. “coach”) and preschool staff evaluation of action plan, including assessing children’s individual goal attainment using goal attainment scale (Kiresuk & Sherman, 1968)	To evaluate child goal attainment, other quality aspects of the preschool program, and to plan for maintenance of preschool program improvements	1–2 h

### Recruitment and Participants

All participating respondents in the present study were recruited from the experimental group in the previously mentioned trial study focused on the implementation of quality improvement actions derived from APERS-P-SE in the Swedish preschool support system (Bejnö et al., 2021). In that study, three cohorts of preschools pertaining autistic children who had recently been enrolled in an EIBI program were recruited within the Stockholm catchment area between September 2017 and June 2019. All respondents in the present study were stakeholders from the experimental condition in the first cohort, with one exception. Habilitation supervisors from all cohorts were recruited to provide a heterogenous set of experiences with EIBI and supervision (i.e., first cohort supervisors were very homogeneous in terms of their extensive experience with EIBI).

In total 19 respondents participated in 13 individual interviews, one involving two parents, and a focus group interview including four habilitation supervisors (see Table 2). Parents of the autistic children were invited to choose whether they both wanted to participate in the interview, or not. In two of the interviews one parent participated, and in one both parents participated. All interviews were conducted in connection with collection of post-intervention data. Respondents received no economical compensation for participating in the study.

**Table 2:** Respondents’ characteristics

Participant	Function	Preschool/supervision experience	Interview format
1	Preschool principal	Data not collected	Individual interview
2	Preschool principal	Data not collected	Individual interview
3	Preschool staff	> 5 years	Individual interview
4	Preschool staff	1–5 years	Individual interview
5	Preschool staff	1–5 years	Individual interview
6	Preschool staff	> 5 years	Individual interview
7	Mother	N/A	Individual interview
8	Mother	N/A	Individual interview
9	Mother	N/A	Joint couple interview
10	Father	N/A	Joint couple interview
11	Supervisor	1–5 years	Individual interview
12	Supervisor	> 5 years	Individual interview
13	Supervisor	> 5 years	Individual interview
14	Supervisor	> 5 years	Individual interview
15	Supervisor	1–5 years	Individual interview
16	Supervisor	1–5 years	Focus group interview
17	Supervisor	< 1 years	Focus group interview
18	Supervisor	< 1 years	Focus group interview
19	Supervisor	1–5 years	Focus group interview

### Interview Guides

A general semi-structured interview guide was generated by members of research team. Derived from this interview guide, four semi-structured interview guides were produced to match each group of respondents (i.e., preschool principals, preschool staff, parents and habilitation supervisors). These guides contained questions on two major topics: (i) key areas and prerequisites for autistic children in Swedish preschool who receive EIBI to support their development, and (ii) evaluation of participating in the APERS-P-SE-based model. Follow-up questions and probes were used when needed to obtain a deeper understanding of topics addressed in the interviews. For more detailed information on the different interview guidelines, see Table 3.

**Table 3:** Interview topics with examples of follow-up questions

Topic	Example of preschool leader interview guide	Example of preschool staff interview guide	Example of parent interview guide	Example of supervisor interview guide
Preschool prerequisites	“What do you believe are the most important aspects of providing a good program for children with ASD in your preschools?” What are the key factors for these children to learn, develop and thrive?”	“What do you believe are the most important aspects of providing a good program for children with ASD in preschools?” “What are the key factors for these children to learn, develop and thrive?”	“What do you believe are the most important aspects of providing a good program for your child in his/her preschool?” “What are the key factors for him/her to learn, develop and thrive?”	“What do you believe are the most important aspects of providing a good program for children with ASD in preschool?” “What are the key factors for these children to learn, develop and thrive?”
EIBI Implementation	“What do you believe are the most important aspects of implementing EIBI for children with ASD in preschools?”	“What do you believe are the most important aspects of implementing EIBI for children with ASD in preschools?”	“What do you believe are the most important aspects of working with EIBI for your child in his/her preschool?”	“What do you believe are the most important aspects of implementing EIBI in preschools?”
APERS-P-SE-Based Model -Perceived changes	“What is your perception concerning your participation in the project? “Have you noticed any changes in how your preschool staff work with autistic children? “Any changes regarding learning environment, inclusion, and/or any new strategies used to promote learning, engagement and participation?” “Have you noticed any changes in the child’s engagement, participation and learning?”	“What is your perceptions concerning your participation in the project? “Changes on how you work with autistic children in due to your participation in project?” “Do you believe that you have gained any new skills on how to promote learning, inclusion, engagement and participation?” “In what way do you believe that your participation might impacted how children with autism function at your preschool?”	“Which changes have you noticed in regard to how preschool staff work with your child? “Changes in your child’s engagement and participation? “Any changes outside the preschool?” “Is there a difference in your collaboration with the preschool now compared to before?” “Do you think that the preschool staff has improved their skills in working with your child?”	”What changes have you noticed in how the preschool staff work with autistic children?” “Have you seen any changes in how the child functions in the preschool that you think can be related to the APERS-based model?” “Do you believe that the APERS-feedback provided to the preschool was relevant, and that it has helped the preschool staff to improve their learning environment and to work with their child with autism?”
APERS-P-SE-Based Model—Content and Structure	“What might have been the most important components?” “What do you think of the in-service training content, and the way the coaching session have been structured?” “What could have been done differently? “Would you recommend the APERS-based model to other preschools?” “Have you experienced any adverse events following your participation?”	“What might have been the most important components?” “What do you think of the in-service training content, and the way the coaching session have been structured?” “What could have been done differently?” “Do you think that you will have use of what you have learnt now in the future?” “Would you recommend the APERS-based model to other preschools?” “Have you experienced any adverse events following your participation?”		“What do you think of the in-service training content, and the way the coaching session were structured?” “What could have been done differently?” “What was the most positive aspect of participating in this project? “Were there any negative aspects?” “Would you recommend the APERS-based model to other habilitation centers, preschools and preschool staff?”

### Design and Procedure

The current study utilized a qualitative design (Patton, 2015) to explore the opinions, experiences and perspectives of four groups of stakeholders on supporting autistic children in the Swedish preschool, and on receiving the APERS-P-SE-based intervention. All interviewers had various previous experience in the field as researchers and clinical psychologists. Duration of interviews varied between 31 and 62 min with an average duration of 57 min for the preschool principals, 51 min for the habilitation supervisors, 39 min for the parents, and 50 min for the preschool staff. The focus group interview was 56 min long.

### Data Analysis

Interviews were recorded with a digital audio recorder, and later transcribed verbatim. Coding and further processing of the data was conducted with NVIVO 12.0 (*NVivo 12, QSR International.*, 2020). Thematic analysis (Braun & Clarke, 2006) was used to analyze all verbal data. A matrix analysis (Miles et al., 2014) portrayed themes for the different stakeholder groups (see Table 4).

**Table 4:**
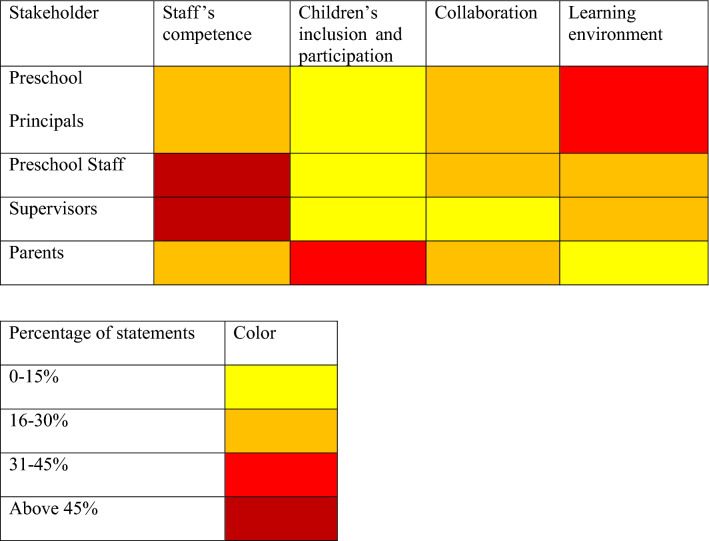
Emphasis of coded statements assigned to themes for each stakeholder group

In order to answer the study’s two research questions, an inductive approach was applied. This allowed the authors to perform the data analysis based on the participants experiences, and through thorough readings of the transcribed verbal data to assign codes to segments of text, thus producing concepts and themes (Azungah, 2018). To promote trustworthiness of identified themes, both the first author of the current study (H.B), as well as the third author (N.L., master student in special education with clinical expertise in autism and EIBI), independently followed the stepwise procedure outlined by Braun and Clarke (2006). That is, first, all interviews were thoroughly read several times and initial thoughts and ideas generated. Second, codes were generated for text units that were of interest for the research questions. Third, all coded blocks of texts were analyzed and preliminary themes identified. Fourth, the themes were matched both with coded units of text and with the rest of the original interviews. Subsequently, general themes were identified and named. Then, for all interviews except for the interviews with habilitation supervisors (here for ethical reasons, as the master student also worked as a senior clinical habilitation supervisor, only the first author did the thematic analysis of that data), the two independent coders assessed intercoder agreement on identified themes, re-evaluated themes, and reached consensus on final themes and names. Finally, for the majority (66%) of interviews with habilitation supervisors, the procedure outlined above was replicated by the last author of the current study (L.R.P) together with the first author (H.B), to further promote trustworthiness of the findings. Subsequently, one of the themes was renamed. Finally, the summary and analysis of data was agreed on by all members of the research team.

To analyze and assess saturation, an inductive thematic saturation approach was applied. With such an approach, saturation is confined to the level of analysis, focusing on the lack of emergence or construction of new codes or themes in the analysis to indicate saturation (Saunders et al., 2018). Saturation was calculated through the use of an emerging codebook (Hennink et al., 2017), and data collection was discontinued when no new codes or themes were identified in the transcribed verbal data.

### Author Positionality

In all qualitative research, it is important to both acknowledge and discuss the positionality of the researchers conducting the study (Braun & Clarke, 2019). In the current study, the research team represented both researchers and clinicians within the field of child- and youth psychiatry, special education, and psychology, with in-depth knowledge of ABA and EIBI, as well as clinical experience in habilitation and preschool. Thus, the interpretations and analyses were influenced by various academical backgrounds and practical experiences, reflecting the complexity and multi-professionality defining the topic of early intervention and preschool program quality for autistic children.

## Results

For the two research questions, four themes across stakeholders were generated: Staff’s competence, children’s inclusion and participation, collaboration, and learning environment. In all interviews, one or more statements were assigned to each identified theme. As can be seen in Table 4, most coded text units were assigned to staff’s competence, which was also the most prevalent theme for preschool staff and habilitation supervisors. The most text units for the preschool principals were assigned to learning environment, whereas for the parents most coded text units concerned children’s inclusion and participation.

In the following section, each theme with corresponding sub-themes is described from the different stakeholders’ point of view. First, the results concerning what the respondents perceived to be the key factors for providing a high-quality preschool program for autistic children obtaining EIBI are presented (i.e., first research question). Second, the results concerning how the respondents perceived that the APERS-P-SE-based model had influenced the program quality are presented (i.e., second research question). Quotations are used to give voice to participants’ experiences and opinions. An overview of the different perspectives among the stakeholder groups can be found in Table 5.

**Table 5:** Summary of results in regard to the study’s two research questions (1 = key factors for autistic children receiving EIBI, 2 = intervention’s influence on preschool program), and stakeholders’ different perspectives on themes

Theme	Preschool principal	Preschool staff	Habilitation supervisors	Parents
*Staff’s competence*	(1) Importance of higher education (2) Intervention helpful for the preschool as a whole. Increased knowledge and self-confidence among staff	(1) Skills and experience needed (2) Improved skills in individualizing support and identifying children’s needs. Increased knowledge, confidence, and independence in providing support. On-site coaching instrumental in building up competence	(1) Specific knowledge about ASD and adaptions important (2) Preschool staff more motivated, better at scaling children’s goals. Sometimes unforeseen burden on supervisors to promote change	(1) Staff need to be able to identify children’s strengths and limitations, and use children’s interests in activities (2) Increased pride among preschool staff due to more knowledge. Less burden on parents to make sure that their child gets the right support
*Children’s inclusion*	(1) Important for children’s development. Adaptations needed. Avoid isolation of child and paraprofessional (2) Inclusion beneficial for all children. Had supported autistic children in forming friendships and learning more social skills	(1) Include autistic child in activities, explain EIBI procedures for other staff. Other children important role-models (2) Work more with peer-mediation and activities involving typically developed peers. Child now playing with peers	(1) Not emphasized (2) Improvements made overall in preschools, however staff-turnover and low engagement negative effect. Most children spending more time with peers, improving communication and social skills	(1) Important for their child to feel like a part of the group, and to avoid stigma. Other children important for learning (2) Child takes more initatives to communicate. Spends more time in activities with peers in preschool, and has better support
*Collaboration*	(1) Collaboration with parents important to provide individualized support to child. More preschool staff need to be engaged in child to promote intervention sustainability (2) Led to collaboration among preschool staff in providing support and EIBI instruction. Positive with more collaboration with habilitation supervisor	(1) Important to build a trustful relation with parents. Collaboration among preschool staff necessary to align general education plan with individualized instruction, co-plan activities, and support goal attainment for the child (2) Helpful to be two preschool staff that can adapt learning environment, and discuss with eachother. Decreased burden on paraprofessional, and improved EIBI sustainability. Less worry for parents that the child will not get adequate support	(1) Important with collaboration among preschool staff to avoid paraprofessional becoming isolated. High level of turnover in preschool very problematic (2) More collaboration among preschool staff, less isolation of paraprofessionals, and easier for children to generalize new skills. Sometimes difficult with collaboration among preschool staff due to staff turnover and low engagement. Preschool staff initially uncertain about their role, and passive in collaboration with supervisors	(1) Important with good relation to preschool staff, to discuss children’s challenges and progress. Important for several staff in preschool to have knowledge and engagement in their child if one or more is away from work (2) Increased collaboration and synchronization of supports among preschool staff. More staff engaged in child
*Learning environment*	(1) Paraprofessional instrumental in preschool. Adapting group size. Clearly defined structure important for children to know what is expected of them, with predictable routines for the child to feel safe (2) Adaptations made in regard to smaller group size, clearer structure, restructure physical environments, and involving peers	(1) Important with paraprofessional and adapted group size and providing structure and support (2) Adaptations made in regard to smaller group size, clearer structure, restructure physical environments, involving peers more. Easier for the child to participate and learn in calmer environment. Visual supports a “simple thing” and yet helpful for all children	(1) Preschools do not typically make adaptions for children with special needs, including ASD. Supervisor has limited insight in the learning environment of children who receive EIBI (2) Adaptations made in regard to smaller group size, clearer structure, restructure physical environments, involving peers more. In one preschool all classrooms reorganized into smaller groups, with more qualified staff for children with special needs. Helpful to be able to be on-site to discuss physical learning environment and implement visusal supports	(1) Make environment autism friendly. Limit group size. Paraprofessional necessary to support the child (2) Adaptations made in regard to smaller group size, clearer structure, restructure physical environments, involving peers more. Easier for the child to understand the activity with less external stimuli. Visual supports implemented, provides a sense of security for child, and helps with communication

### Staff’s Competence

#### Key Factors

##### Importance of Education and Training Related to Autism

All respondents highlighted the importance of knowledge about autism and accommodating autistic children in preschool classes as instrumental for promoting their development. The preschool principals especially noted the importance of a higher education background among the preschool staff to understand the terms and language used by the habilitation supervisors. However, they also described that they have limited funds for recruiting paraprofessionals for autistic children, noting that they usually have to employ someone lacking experience and education on working with autistic children. One principal stated:

With the EIBI programs, the things that you need to learn and use in your work can be quite advanced. You need to have staff with knowledge about children, their needs, and their development, I would say, to be able to support them in the best way. That is really important […] but my experience is that typically, someone young, without any education, gets to do the job.

The importance of preschool staff competence was also echoed by the habilitation supervisors, who described that while preschool staff may have substantial experience and knowledge concerning mainstream preschool pedagogy, there is a need for increased knowledge about how to adapt the learning environment to non-typically developed children. They also highlighted the importance of individualizing supervision as some may learn new skills swiftly, while others may need a lot of support.

The interviewed parents specifically mentioned the need for preschool staff being able to identify both strengths and limitations of their child, and also to utilize their child’s interests to engage them in new activities and support them in the right direction.

#### Perceptions of Intervention on Staff’s Competence

##### Improving Competence, Self-confidence and Interest

Stakeholders were in consensus that the APERS-P-SE-based model had helped preschool staff become more knowledgeable and confident. One principal described how the intervention had been very helpful not only for the child, but for the preschool as a whole:

We didn’t really know what to do with this child when he was enrolled in one of our groups, to be honest. I didn’t have much experience myself, like the rest of us here. So, first receiving the EIBI, and then being part of this research project has helped us a lot. Not only the child, but for the entire preschool, and our knowledge and competence, I would say.

Preschool principals noted a higher level of knowledge, self-confidence and engagement in working with the autistic child among staff in other groups in the preschool. For example, the principals described that preschool staff from other groups participated in discussions about the child’s progress during staff meetings, that they now greeted the autistic child in the morning, and that they engaged with him during outdoor recess where children from different preschool groups meet and play together on the playground.

The preschool staff working directly with the children specifically mentioned increased skills in identifying children’s skill deficits compared to typically developed peers, and in planning for providing evidence-based instructions based on the identified needs of the individual child. Gaining more understanding of the function of children’s interfering behaviors, and changing the way they acted in regards to those behaviors by identifying important contextual factors, was also brought up. Several preschool staff described that the most important experience from the research project had been to gain new knowledge, thus becoming more independent, and taking more initiatives on their own in supporting the children. Additionally, they stated that they could use competencies obtained through the research project with other children than the ones receiving EIBI:

I use what I have learnt from this research project with other children as well... In the circle time, for example. Or how to participate in the play time…. I think that it has helped me a lot, really, not only to support him, but also with the other children.

Parents described an increased level of interest, engagement and involvement from the preschool staff in their children compared to before. One mentioned that she had the impression that the preschool staff showed more pride in their work when they saw that the things they did made a difference for the child. Another parent described that the increased competencies of preschool staff lessened the burden on her:

Previously I felt like the ‘project leader’, who had to make sure that they actually made adaptions to accommodate and support my child. Thanks to this research project, I don’t feel like I need to do that to the same extent […], they improve their competence now because they want to, not because someone else tells them to. It feels great to see their willingness to engage.

Habilitation supervisors specifically noted that in general preschool staff had become more skilled in goal setting, that they became more actively involved with the autistic child during the course of the intervention, and several noted increased motivation and confidence on behalf of preschool staff. However, some also experienced that preschool staff initially had difficulties in specifying goals, entailing that in some cases increased and unforeseen burden was placed on them to push preschool staff to promote changes in the learning environment, define goals, and methods. Also, the supervisors hypothesized that possibly due to lack of previous knowledge and skills, some preschool staff also appeared to lack motivation.

##### On-Site Coaching as a Means for Learning

One instrumental aspect of the intervention to improve competence highlighted by the preschool staff was the importance of receiving on-site coaching. Preschool staff believed that the coaching had a much greater impact when provided on-site, compared to supervision at the habilitation center. One staff member stated:

I think that I learnt the most by practicing giving instruction in the preschool setting. I do it while the coach is observing me and the children, and seeing things that I don’t notice in that very situation. I just do it without reflecting about it. And then we take a step back, and I’m provided with feedback. ‘Try doing this instead’… . And then you try again.

Furthermore, preschool staff mentioned that it would have been even better if all staff would have been able to participate in the research project and receive coaching. However, some habilitation supervisors described perceiving themselves as lacking sufficient skills and experience in promoting naturalistic evidence-based practices in regular preschool activities, causing them to feel uncomfortable. With regard to peer-mediated intervention:

It became more difficult for me (to coach) when I decided to implement peer-mediated intervention, which I had not used before. I tried to go through my documentation to learn more about how to plan for and structure it. It became obvious for me that if I didn’t feel comfortable with the method as a supervisor, it became much harder to teach it to the preschool staff.

### Children’s Inclusion and Participation

#### Key Factors

##### Adapting Activities for Inclusion

The preschool principals described that inclusive preschool groups allows for the best potential development for autistic children in the preschool. However, this entails several different adjustments that needs to be done in the preschool setting. Preschool activities need to be adapted to fit the needs of all children, including autistic children. The preschool staff also has to make sure to avoid the risk of the paraprofessional becoming isolated with the child outside of the rest of the preschool group, which subsequently means that all of the preschool staff need to be engaged in working with the autistic child and provide instruction. One interviewed preschool staff described that one way of making the other staff more engaged in “her” child could be to actively include the autistic child in the other children’s activities on a daily basis, and to explain the EIBI procedures and principles for them. Parents also highlighted the importance of inclusion for their child, and the wish for them to be regarded as a part of the group. They described that they wanted the preschool staff to offer the opportunity for their child to participate in all activities, to not limit them and give them a feeling of being different than the others.

##### Importance of Peers

Several preschool staff mentioned that other children can function as important role models, by modeling skills, and providing peer-support. However, also noted was that other children may hinder inclusion if they display a disinterest for the autistic child, and do not make any attempt to initiate contact with him or her: “In his former group, the other children were less interested in him. When they didn’t take initiatives to play with him, he became more ‘left out’”. One preschool staff perceived providing EIBI instruction in a smaller group as much more rewarding and engaging for the autistic child, compared to one-to-one instruction sessions: “Her day gets so much better this way […] She can tag along and be like any other child. Not just a lot of things she needs to do. She too gets to join the other children in their play”. One parent shared the impression of the importance of other children as role models for her child, and stressed the need for implementing EIBI together with peers so that her son could imitate other children rather than preschool staff, and improve his communication and social skills. The habilitation supervisors did not to the same extent highlight inclusion and participation as key features for autistic children to develop and thrive in their preschool.

#### Perceptions of Intervention on Inclusion and Participation

##### Impact on How Preschools Work

Preschool principals were in agreement that participation in the research project had led to improvements in working with inclusion. It had been beneficial for both the autistic children, and the other children in the preschool group, leading to acquisition of social skills and new friendships:

Yes, we have tried to arrange more activities for the autistic child together with other children. And we have seen over and over that this is really helpful in two ways, not just only for him, but also for the other children to get to know him more. So that he can go from someone who may cause trouble and interrupt circle time, to someone that you can play with.

Preschool staff discussed the importance of inclusion and social interaction and particularly the relevance of peer-mediated instructions as a part of the research project. One staff member stated:

I mean, we have been working with peer-mediated instruction, and I think it’s a way to get him more engaged and included. So, instead of being in a separate room, he’s now actually playing with his friends while he is provided with instruction and learning new things.

However, positive changes concerning inclusion and participation were not found in all preschools. One habilitation supervisor stated that she could see no improvements in these areas for the autistic child due to low engagement of preschool staff, as well as high staff turnover.

##### Impact on Children

The preschool principals maintained that the autistic children had made substantial improvements during the course of the intervention, especially regarding social- and communicative skill development. They believed this to be related to their participation in the research project, and the increased emphasis of supporting the children’s inclusion and participation in the preschool activities, compared to the regular EIBI implementation set-up.

All parents described their children as taking more initiatives to communicate, with more children and preschool staff, and attributed many of the children’s newly acquired skills in communication and social interaction with their peers to the preschool staff’s improved skills in supporting their children in inclusive settings. As a result, they saw that instead of keeping to themselves, their children participated more in preschool activities, and communicated more with the other children. One parent described it in this way:

We see changes. When we visit friends, he seeks to communicate with their children. In the past, only “Sara” (the paraprofessional) worked and communicated with him. But now, the other educators greet and include him in the group. It's wonderful to see…

For the habilitation supervisors, the intervention was described as having a significant impact on the inclusion and participation of the autistic child. They mentioned observing increased social interaction and communication with peers, improvement in play skills, and substantially more time spent together with other children compared to before the intervention, in activities such as play time, circle time, and when eating lunch.

### Collaboration

#### Key Factors

##### Collaboration Between Preschool and Parents

Preschool principals underscored the importance of having a good relationship and collaboration with the autistic children’s parents as being crucial to provide appropriate supports for the child. The preschool staff agreed on the importance of having a trustful relation with the child’s parents, for the parents to be able to rely on preschool for implementing the program and providing support on a daily basis. Preschool staff members and parents mutually agreed on the necessity of having a close partnership, to work on the same goals at home and in the preschools. For parents this included for both them and the preschool staff to be honest with each other, to discuss and address difficult situations and challenging topics, and not only talk about things are running smoothly.

##### Collaboration Among Preschool Staff

All stakeholder groups agreed on the importance of collaboration among preschool staff to support the autistic child. Preschool teachers brought up the importance of aligning the general education of the preschool with the individual goals for the autistic child. This entails a close collaboration with the habilitation supervisor who needed to make sure that the interventions and procedures for the child can be adjusted to the natural environment of the child in the preschool. Also, this involved joint planning between paraprofessionals and preschool teachers to include the autistic child in preschool activities. One preschool teacher stated: “To always consider how this or that general activity will work for him when they do the planning, what may be challenging, what may work better, when will we need to adapt the group size of children”. Another aspect of the collaboration between the preschool staff was the importance of all preschool staff in child’s group being informed about what the individual goals were for the child. Lacking this knowledge may lead to some preschool staff not placing any demands on the child, thus not giving the child any possibilities to practice his or her newly acquired skills.

Parents and habilitation supervisors also underscored the importance of having as many preschool staff as possible engaged in the child’s individual EIBI program, to avoid the fragile situation of only one preschool staff having the knowledge, engagement and know-how, thus threatening intervention sustainability. In keeping with statements above, one of the habilitation supervisors described the high level of overall staff turnover in the Swedish preschool as being very problematic. Habilitation supervisors also mentioned the importance of collaboration between the preschool staff to prevent isolation of the paraprofessional: “Most paraprofessionals I meet really enjoy implementing EIBI, but they also feel really lonely at the preschool. And there is not always a great understanding among all the colleagues at the preschool about what they are doing.”

#### Perceptions of Intervention and Collaboration

##### Improved Collaboration Among Preschool Staff

All stakeholder groups shared the general impression that collaboration among preschool staff had been improved during the course of the research project. Preschool principals described more collaboration in providing the EIBI program, and overall support for the child. The preschool staff brought up that it was helpful to have two preschool staff collaborating in implementing the EIBI program, making adaptions in the learning environment, and supporting the child, as compared to one before. Specifically, to be able to reflect and discuss strategies with each other, to plan for the child to generalize new skills, and taking turns in providing instruction was highlighted. This meant a lower probability of jeopardizing the EIBI program and the overall support of the child, if one of the preschool staff became ill or left the preschool for another job. It also entailed a decreased individual burden on the paraprofessional. Additionally, the preschool staff described that they had perceived the children’s parents as having an increased feeling of security about their children in the preschool, when they could see that more preschool staff collaborated in supporting their child:

If the other preschool staff (the ones not actively involved in the study) have a better understanding of how to do things, then they show you more respect, and they may also dare to engage in more interaction with the autistic child, themselves.

The parents described that the preschools participation in the project had led to increased collaboration among preschool staff, leading to better coordination, spreading of knowledge, a more coherent focus on the EIBI instruction in the preschool setting with more people involved, as well as a more synchronized approach to their children: “Previously, only the paraprofessional used to work with him and communicate with him. Now, if she is not there when he arrives, other preschool staff come out to welcome him and include him in their group of children.”

Most habilitation supervisors agreed that the research projects’ increased focus on involving more preschool staff with the child had led to more collaboration between preschool staff, less isolation of the paraprofessional, and subsequently improved generalization of the children’s newly acquired skills.

##### Uncertainty About Roles and Responsibility in Collaboration

A few habilitation supervisors, however, described difficulties in collaboration amongst preschool staff and in obtaining joint on-site coaching, mostly due to extensive sick leave, staff turnover, and sometimes lack of interest and limited resources (i.e., time). Furthermore, in regard to collaboration between habilitation and preschool within the research project, some supervisors also experienced that preschool staff seemed uncertain about their roles and responsibility, taking a passive stance, expecting the supervisors to take responsibility for tasks that were the preschools responsibility, such as defining inclusive goals. This was perceived as negatively influencing the impact of the APERS-P-SE based model, and thus the learning environment for autistic children. In contrast, preschool principals noted that the research project had led to increased collaboration with the habilitation supervisor in general, which they deemed as beneficial for the autistic child.

### Learning Environment

#### Key Factors

##### Importance of Paraprofessional

In the autistic child’s immediate learning environment, all stakeholders described having a paraprofessional as a key prerequisite for the learning and development of the child. The paraprofessional is needed to both receive in-service training and EIBI supervision, implement interventions, and as an overall support. One principal stated: “I think it as an absolute prerequisite for this child to have a designated paraprofessional, to be able to attend this preschool in a positive way”.

##### Adapting Group Size

All stakeholder groups underscored the importance of adapting group size, for example by dividing larger groups of children to smaller groups, in certain activities. This was perceived as beneficial for children in general, and for autistic children in particular. Adapting group size entails less noisy settings, with less social relations in place, and thus a slower pace in the activity. This can make the children calmer, help autistic children to initiate contact with other children in the group, and make it easier for paraprofessionals to provide learning opportunities for the child, compared to being in a bigger group.

##### Adapting Physical Environment and Structure

All stakeholder groups agreed on the importance of adapting the physical environment, to make it structured and autism friendly. For example, preschools principals described a clearly defined structure in the preschool where both preschool staff and children know what’s expected of them, with a familiar environment, and predictable routines, as essential for the children to feel safe in the preschool environment. However, they also pointed out that it places demands on the physical environment, as well as thorough planning: “You need enough space, physical delimitations, and to know exactly who will be doing what during the day among the staff”. Also, as an additional benefit, with a clearly defined structure, the preschool becomes less vulnerable for staff turnover, and other changes that might come up.

In contrast to the preschool principals, all of the preschool staff brought up the importance of using visual supports. When striving for structure and supportive routines, to make the children less anxious, visual supports can be used both to show children and preschool staff how things are to be done. Habilitation supervisors described an overall impression that many preschools in Sweden do not make adaptations in the learning environment for autistic children. Specifically, lack of structure, big open spaces, lack of clear instructions and low expectations on the child was mentioned. However, although occasional visits to the preschool may occur to plan for where in the preschool one-to-one EIBI instruction can be provided, or to provide supervision in a separate room, supervisors described a limited insight among staff in general concerning the importance of the preschool learning environment for autistic children who receive EIBI.

#### Perceptions of Intervention and the Learning Environment

##### Improving Learning Environment

All stakeholder groups perceived that as a result of the project staff had adapted the learning environment in order for the autistic child to be able to participate and learn. This concerned working with smaller groups to limit distracting stimuli, restructuring the physical environment, and using other children in the preschool group as peer buddies. Also described was improved structure in specific activities, with clearer instructions, using task-analysis, visual supports, and connecting children’s individual goals (e.g. increased independence in the preschool setting) with the physical preschool environment (e.g. implementing more visual supports). One of the preschool staff provided the following example in regard to limiting group size and external stimuli:

We have now introduced a mini-format circle time with him, where he can practice turn-taking with the other children and listen more to what they say. You can introduce a game in the mini-circle time as it is calmer, and it is much easier for him to participate and concentrate, compared to our ordinary circle-time.

From a parental perspective, one mother described that when activities became easier to understand it made it easier for her child to engage. She also had the impression that participation in the project had been beneficial not only for her child but for the other children as well. Accordingly, one habilitation supervisor described that in one of the preschools, all preschool classrooms were reorganized into smaller groups, with the majority of the more experienced and qualified staff assigned to the classroom where most children with special needs were enrolled.

##### Implementing Visual Supports

All stakeholder groups brought up the importance of implementing visual supports, within the framework of the APERS-P-SE-based model.

For example, one preschool staff shared her experience of implementing visual supports in the preschool setting like this: “It (visual supports) has been a great support. You almost feel bad about not having introduced it earlier, also for the other children without autism. It is such a simple thing, really.” In regard to perceiving clearer instructions, and improved routines, one parent had likewise especially noted the implementation of visual supports:

Now there is a visual support showing my child where she can sit during lunch. It is like a routine. It (visual supports) is something that gives her a sense of security, to know that “this where I sit”. But also, for communication with pictures, we have noted that it (her communication) has become much, much better.

From the habilitation supervisors’ perspective, one supervisor highlighted visual supports as an example of the usefulness of being on-site and more specific about how the preschool staff could improve the learning environment:

For example, we were working on one goal about how to use visual supports in the preschool. And many times before had it mostly been me sitting somewhere else [supervisor’s office] talking about how things could be done. But this time, I actually walked through the different settings with the preschool staff, and together, we discussed what we could do.

However, another supervisor experienced that in the preschool that she supervised, although she had tried a similar approach, the preschool staff still seemed to struggle with how to actually use the visual supports.

## Discussion

The current study used a qualitative design to explore the views and experiences among four groups of stakeholders about (1) what they perceived as key features for providing a high-quality preschool program for autistic children who are receiving EIBI to promote optimal development, and (2) how they perceived that the APERS-P-SE-based intervention had influenced the preschool program quality for the autistic child. To the authors best knowledge, this is the first study examining different stakeholders’ views on providing a high-quality preschool program for autistic children in Sweden. It is also the first qualitative study exploring participants’ experiences following participation in an APERS-P-SE-based model of professional development, with stakeholders generally describing the positive influences of the model. Previous studies from Sweden (Bejnö et al., 2021) and the U.S. (Odom et al., 2013; Sam et al., 2020) have demonstrated promising results in improving the program quality for autistic children using quantitative outcome measures. However, a qualitative evaluation could as previously noted both complement, enrich and provide a deeper understanding of how such a model is perceived among stakeholders (Cleland, 2017).

In regard to the first research questions, findings from interviews with stakeholders suggest that the key features of a high-quality preschool program for autistic children include high levels of *competence* among preschool and habilitation staff, structured and systematic work in supporting *children’s inclusion and participation*, comprehensive *collaboration* between preschool staff as well as with parents, and an active and systematic approach in adapting the immediate *learning environment* to the needs of autistic children. Specifically, paraprofessionals and preschool teachers collaborating and working with all children (i.e., team teaching or co-teaching; Friend et al., 2010), including autistic children in activities with typically developed peers, providing structure, limiting group size, and providing peer-support or peer-mediated instruction, can be highlighted as specifically important findings.

The current findings corroborate with findings from other studies emphasizing the need for competence among preschool staff and in-depth knowledge and skills among supervisors (Ala’i-Rosales et al., 2010; Eikeseth et al., 2012; Guldberg et al., 2011; Kendall et al., 2013; Leaf et al., 2016; Scheuermann et al., 2003; Stanford et al., 2020) which maps onto points identified in staff’s competence such as knowledge, skills, education and experience. Previous research has also identified the need for close collaboration between preschool and parents to promote intra-professional collaboration (Fallon & Zhang, 2013; Guldberg et al., 2011), and the importance of making adjustments in the social and physical environment and processes of care (CAST, 2008; Kendall et al., 2013; Krieger et al., 2018; Odom et al., 2013, 2018; Piller & Pfeiffer, 2016) also maps onto themes of collaboration, and learning environment. Additionally, previous research has also identified the importance of peer-interaction, peer-mediated intervention, and promoting participation (Guldberg et al., 2011; Gunning et al., 2019; Krieger et al., 2018; Little et al., 2014; Odom, 2019; Piller & Pfeiffer, 2016), which maps onto different aspects of children’s inclusion and participation.

Stakeholders in the current study differed somewhat in their emphasis on the most important features within the four themes, depending maybe on their different perspectives and roles (See Table 4 for a visualization of the “temperature” of each theme, and Table 5 for a brief summary of how the perspectives varied). For example, the habilitation supervisors mainly described the competence needed from the preschool staff to implement the EIBI program, not emphasizing inclusion to the same extent as other stakeholder groups. This may potentially be explained by the fact that supervisors do not typically spend much time in the preschool setting. Instead, their area of response within the Swedish early intervention system and EIBI model is to be responsible for the individualized EIBI program, with almost all supervision provided at the habilitation center, with one-to-one instruction between an adult and the child. In contrast, the interviewed parents spoke more about the importance of including their children in their preschool group, and making them feel like they were a part of that group. The preschool principals focused more on the learning environment, how to organize groups, arrange the physical environment, provide a structure, recruit skilled staff, etc., whereas the preschool staff focused more on their own competence, and in their collaboration with each other.

In regard to the second research question, our findings suggest that the APERS-P-SE-based model was believed to substantially improve the abovementioned preschool program quality features. Thus, the perception of the stakeholders converge with the quantitatively measured outcomes in the quasi-randomized group study conducted by Bejnö et al. (2021), and further support and nuance the social validity (Wolf, 1978) of the APERS-P-SE-based model, suggesting that it is feasible to integrate an APERS-based model in the Swedish support system. In particular, two notable changes were described by stakeholders as resulting from participation in study. Firstly, several of the children were described by both staff and parents being less in own world, engaging more with staff, as well as more peers being involved with the child (i.e., not just the paraprofessional). Secondly, staff reported feeling more self-confident. This may be of specific importance as low self-efficacy and high job-demands among teachers has been associated with signs of burnout, which in its turn is associated with sick leave, and teachers leaving the profession (Arvidsson et al., 2019). More preschool staff involved in the autistic child may also be of particular importance for intervention sustainability and children’s overall support, as the preschool in Sweden is known as an educational context with high levels of staff attrition (Sveriges Radio, 2018). Furthermore, and importantly, the APERS-P-SE-based model was described as being helpful not only for autistic children, but for all children in the preschool.

Concerning the different components of the model, on-site coaching (Kucharczyk et al., 2012) based on initial APERS-P-SE assessments seemed to have had the most significant effect. It was described as essential in promoting many of the described changes following the APERS-P-SE-based model; improving the preschool staff’s practical skills in providing instruction, goal setting and increasing children’s inclusion and participation, and in adapting the immediate physical learning environment to the needs of the autistic children. It also allowed more preschool staff to obtain support from the habilitation supervisors, compared to the typical situation in which one member of staff (often paraprofessional) travels to a habilitation center to receive EIBI supervision. However, it was also suggested that involving more (i.e., all) staff in the research project would have been more beneficial. The onsite coaching involving more members of staff appears to have enhanced collaboration between preschool staff in providing support and instruction to the autistic child. Indeed, Fixsen et al. (2009) describe coaching, following introductory training, as the main tool to bring about behavior change among practitioners, and to implement and maintain evidence-based practices- and programs. An unforeseen finding was that several habilitation supervisors described it as challenging to provide on-site coaching, both due to limited knowledge and lack of previous experience in embedding inclusive focused interventions such as peer-mediation in naturalistic settings, but also due to preschool staffs’ limited knowledge and skills as a prerequisite to benefit from coaching. Both situations are concerning and underscore the importance of staff on all levels obtaining adequate and appropriate training. This is especially relevant considering that previous research has underscored the importance of knowledge about how to individualize and adapt EIBI implementation to the child’s contextual learning environment (Långh et al., 2020). Findings also suggest that there is clearly a need for a more flexible system in which number of onsite coaching sessions vary from once a month to weekly or more.

## Limitations

Although consistent with a qualitative approach, the findings reflect subjective, individual experiences of purposively selected stakeholders and as such limit inferences to this specific context. As occurs with qualitative studies, readers should make a judgement about the applicability of the results to their setting based on a description of the context (Korstjens & Moser, 2018). Interviewing parents, preschool principals and preschool staff from the other six preschools that received the APERS-P-SE-based model could have provided a broader set of perspectives across contexts and possibly a deeper understanding concerning strengths and shortcomings of the project, although it is not clear that this would have improved saturation. Alternatively, participants could have been randomly selected, or all participants could have been interviewed, with half of the interviews randomly selected for analysis. Habilitation supervisors from all groups, however, were interviewed, shedding the light on consequences of variations derived from experience. In sum, these results may serve as an addition and complement to quantitative outcomes, rather than confirming or rejecting an a priori hypothesis. The results should also be considered preliminary, and could in future studies potentially be contrasted or extended with a larger and randomly selected sample of participants, or with complementary methodology such as surveys, based on the themes generated in the current study.

One important limitation of the current study is that some of the interview questions were closed and may have been perceived as leading. For example, a question like “Do you believe that you gained any new skills on how to promote learning, inclusion, engagement and participation?” may have indicated the type of response that was expected from the interviewee, thus potentially creating a bias towards affirming the value of the intervention (Agee, 2009), which should be considered when the study’s results are interpreted. An additional limitation is attrition, reflecting previous data on preschool staff turnover (Sveriges Radio, 2018), and highlighting the importance of implementation sustainability by involving more preschool staff, and providing coaching on-site. In one of the preschools there was no attrition, yet in the other two, only the preschool staff who had not attrited were interviewed. Accordingly, the perspectives of these interviewed individuals may not represent the experiences of the preschool principal and the two preschool staff who did not remain in their preschools during the course of the APERS study. Furthermore, although not necessarily a limitation, it should be noted that no stakeholders were interviewed from the control group in the previously described group study by Bejnö et al. (2021). Thus, the current study does not provide a group comparison, but rather an extension of the results from the group study, in regard to the influence of the APERS-P-SE-based model. Finally, all interviews were conducted at the end of the intervention. Sequencing interviews at the beginning, during, and at the end of the intervention may have provided more information in regard to the process of improving preschool program quality over time, within the framework of the APERS-P-SE-based model.

## Conclusions and Practical Implications

Based on the present findings, implementing EIBI programs for autistic children in inclusive preschool programs should entail carefully adapting procedures and instructions to children’s specific preschool settings, with pertaining inclusive activities. Other children should be involved as much as possible in the provision of instruction, for example through the use of peer-mediated intervention. Furthermore, EIBI programs should include implementation of visual supports in the preschool environment. Such inclusive practices may be supported by professional development, provision of paraprofessional personnel, and coaching from habilitation supervisors in the preschool setting. On the preschool side, group sizes could be adjusted to meet the needs of autistic children, and to promote learning for all children in as many activities as possible. Other adaptations of the learning environment such as physical delineations and visual supports could be implemented to provide structure for all children, including autistic children. Typically developed children may serve as role models and peer-buddies for mutual learning in social interaction and communication. Collaboration and joint responsibility among preschool staff should be encouraged in providing support and instruction to the autistic children, to promote learning for both children and staff. Structures, procedures and activities should be established that are not dependent on an individual preschool staff, to ensure sustainability in practices over time.

Finally, our findings suggest that the evaluated APERS-P-SE-based model, including assessment of preschool program quality for autistic children, in-service training, and on-site coaching, could be used to realize the guidelines suggested above. Thus, providing the prerequisites for successfully implementing evidence-based practices to produce positive outcomes for autistic children (Odom et al., 2013), in line with NPDC’s theory of change. However as noted, this entails providing coaches with adequate training and support to be able to comfortably provide coaching in preschool settings, where focused interventions used within EIBI programs may be embedded in regular routines.
